# Nanoparticle-based detection of foodborne pathogens: Addressing matrix challenges, advances, and future perspectives in food safety^☆^

**DOI:** 10.1016/j.fochx.2025.102696

**Published:** 2025-06-25

**Authors:** Himanshu Jangid, Mitali Panchpuri, Joydeep Dutta, Harish Chandra Joshi, Maman Paul, Arun Karnwal, Akil Ahmad, Mohammed B. Alshammari, Kaizar Hossain, Gaurav Pant, Gaurav Kumar

**Affiliations:** a School of Bioengineering and Biosciences, Lovely Professional University, Jalandhar, Punjab, India; b School of Pharmaceutical and Population Health Informatics, DIT University, Dehradun 248009, Uttarakhand, India; c Department of Chemistry, Graphic Era (Deemed to be University), Dehradun 248002, Uttarakhand, India; d Department of Physiotherapy, Guru Nanak Dev University, Amritsar, Punjab, India; e Department of Microbiology, Graphic Era (Deemed to be University), Dehradun, Uttarakhand, India; f Department of Chemistry, College of Science and Humanities in Al-Kharj, Prince Sattam bin Abdulaziz University, Al-Kharj 11942, Saudi Arabia; g Department of Environmental Science, Asutosh College, University of Calcutta, 92, Shyama Prasad Mukherjee Rd, Bhowanipore, Kolkata 700026, West Bengal, India; h Amity Institute of Microbial Technology, Amity University Rajasthan, 303002, India

**Keywords:** Nanoparticles, Foodborne Pathogens, Food Matrices, Detection Systems, Food Safety, Smart Technologies

## Abstract

Foodborne diseases pose significant public health and economic challenges worldwide, with conventional pathogen detection methods, such as culture-based assays and PCR, often hindered by the complex food matrix in categories like dairy, seafood, fresh produce, and processed foods. These matrices, containing fats, proteins, biofilms, and salts, interfere with detection accuracy, reducing the sensitivity and robustness of traditional approaches. Nanoparticle-based detection systems have emerged as transformative tools to overcome these challenges, offering enhanced sensitivity, rapid detection, and adaptability to real-time monitoring. Gold, silver, magnetic, polymeric, and hybrid nanoparticles leverage their unique optical, magnetic, and functional properties to facilitate specific pathogen identification while mitigating food matrix interference. Recent advancements include nanoparticle-functionalized biosensors, magnetic separation platforms, and smart detection systems integrated with IoT and blockchain for traceability and real-time contamination alerts. However, challenges such as high production costs, regulatory gaps, and scalability hinder their full-scale adoption. This review critically examines matrix-specific adaptations of nanoparticle-based detection technologies, their comparative advantages over traditional methods, and their integration with smart technologies to ensure food safety. Future directions emphasize interdisciplinary collaboration, eco-friendly synthesis, and regulatory frameworks to address commercialization hurdles and revolutionize pathogen detection across the global food industry.

## Highlights

• Nanoparticles enable ultra-sensitive, real-time pathogen detection in complex food matrices.
• IoT and blockchain-integrated nanosensors revolutionize food safety monitoring.
• Hybrid nanomaterials offer scalable, eco-friendly solutions for food safety.
• Regulatory frameworks and interdisciplinary innovations drive nanoparticle adoption.

## 1 Introduction

Foodborne diseases are a critical health issue and bear a heavy cost; annually, tainted food products cause 600 million cases and 420,000 deaths worldwide (Awad et al., 2024). Outbreaks cause human losses and immense economic losses, such as recall, legal procedures, and medical costs. It is critical to promptly and accurately identify the causative pathogens of foodborne diseases to reduce the risks and provide safe food security (Almaary, 2023; Rahman et al., 2023). It is well known that traditional detection methods for pathogens, including culture, enzyme-linked immunosorbent assay (ELISA) and polymerase chain reaction (PCR), are relatively reliable and accurate. However, they are mainly used for processes that usually take longer periods for processing, extensive laboratory equipment, and professional personnel; hence, they are not applicable for site observation or real-time monitoring (Quintela et al., 2022). In addition, when these techniques were applied to complex food matrices, such as dairy, seafood, or processed foods, other issues related to sensitivity and specificity arose. Recently, nanotechnology has emerged as a revolutionary approach to overcoming these limitations (Wang & Duncan, 2017). Nanomaterials were characterized by increased surface-to-volume ratios, tunable surface functionalities, and quantum effects. Such unique properties facilitated the development of highly sensitive, specific, and fast detection platforms. For example, gold nanoparticles have been successfully applied in colorimetric assays for the detection of *Salmonella* and *Listeria monocytogenes,* and can detect up to 10 CFU/mL (Quintela et al., 2019). Similarly, graphene-derived nanostructures allowed the development of field-effect transistors that were capable of real-time pathogen detection within complex food matrices, such as seafood (Thakur et al., 2018). Moreover, electrochemical biosensors with nanomaterials, such as carbon nanotubes and Metal-Organic Frameworks (MOFs), have further enhanced the application of nanotechnology in food safety. These systems generally exhibited high sensitivity and rapid response and were, therefore, suitable for the early warning systems of food supply chains (Harinathan et al., 2024; Malik et al., 2023).

Advancements in aptamer-based sensors and nanozyme-based platforms have shown their multiplex detection ability, where several pathogens can be detected simultaneously, which is a prerequisite for wide industrial applications (Chatterjee et al., 2020). In parallel, cutting-edge molecular technologies such as CRISPR-based detection systems have emerged as powerful alternatives. CRISPR-Cas systems, originally developed for gene editing, have been repurposed into ultra-sensitive biosensors capable of detecting foodborne pathogens with high specificity and speed. Platforms like SHERLOCK and DETECTR can identify bacterial and viral DNA/RNA in complex food matrices, often within an hour, without the need for advanced thermal cycling equipment (Mustafa & Makhawi, 2020) (Gootenberg et al., 2017). These systems complement nanoparticle-based biosensors and provide a promising direction for multiplex, point-of-care pathogen detection in food safety applications. Despite these innovations, much remains to be surmounted. In most cases, the inherent complexities in matrices-food, with widely dispersed interference agents such as proteins and lipids, frequently interfere with nanoparticle sensing-based systems (Rodriguez-Quijada et al., 2020). Second, issues facing mass production due to problems of environmental toxicity along with regulatory compliance and potential unacceptability on a commercial scale have confined this set of technologies to date. This review therefore explored the detection technologies involved in nanoparticles used in identifying foodborne pathogens by discussing their applications in diverse food matrices and relative advantages over traditional methods. On the other hand, it investigated extant challenges and identified probable avenues for future incorporation with advanced systems, such as AI and blockchain, for the improved monitoring of food safety. This study showed that nanotechnology can revolutionally impact food safety practices, so many laboratory innovations have been implemented to direct industry implementation (Alafeef et al., 2020; Park & You, 2023; Xiao, Li and Xu, 2022a, Xiao, Li and Xu, 2022b).

## 2 Advancements in nanoparticle-based detection platforms

**Fig. 1:**
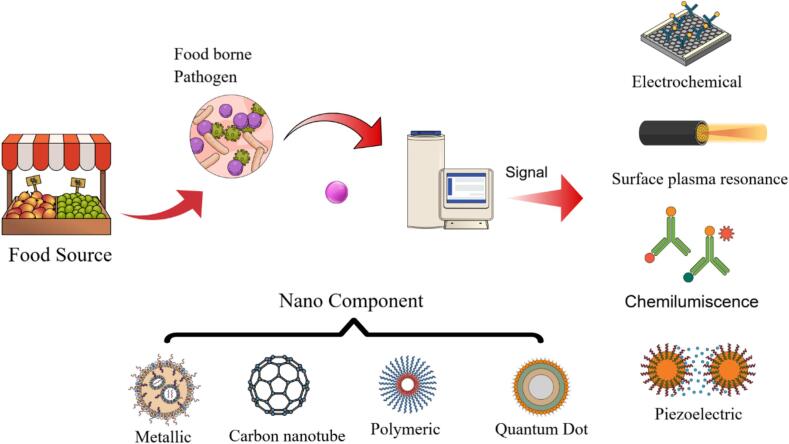
Schematic representation of nanoparticle-based biosensors for foodborne pathogen detection. The process begins with pathogens from a food source, followed by signal generation and detection using various mechanisms. Nanomaterial components include metallic nanoparticles, carbon nanotubes, polymeric nanoparticles, and quantum dots, while detection platforms incorporate electrochemical sensors, surface plasmon resonance, chemiluminescence, and piezoelectric methods for accurate and sensitive analysis.

Foodborne infections and pathogens are now identified using nanotechnology-based techniques that provide unparalleled sensitivity, specificity, and detection speed. Compared to these nanotechnology-based platforms, the traditional culture-based method and PCR have a significant disadvantage: they are time-consuming, labour-intensive, and often require technical expertise. The use of nanoparticles provides an opportunity to design novel biosensors and diagnostic devices by combining molecular recognition at the nanoscale with signal amplification. These platforms exploit the unique physicochemical properties of nanoparticles, such as high surface area, tunable optical properties, and adaptive functionality, to enhance detection sensitivity, even at very low concentrations of pathogens. Metal-based, carbon-based, polymeric, and hybrid nanomaterials constitute some of the most prominent contributors across the categories of nanoparticle types, each offering the unique advantages of sensitivity and applicability (Du et al., 2022; Sohrabi et al., 2022; Zaid et al., 2019). Schematic illustration (Figure 1) of nanoparticle biosensors for the detection of foodborne pathogens. Schemes include a pathogen from the food source, a signaling event, and the read-out of that signal from mechanisms. Nanomaterial parts include metallic nanoparticles (NPs), carbon nanotubes, polymeric NPs, and quantum dots whereas electrochemical sensors and surface plasmon resonance Chemiluminescence along piezoelectric are used as the devices of detection for better sensitive and accurate analysis.

### 2.1 Metal-based nanoparticles

Metallic nanoparticles (MNP), such as gold nanoparticles (AuNPs), silver nanoparticles (AgNPs), and copper-based nanomaterials, have emerged as prominent probes for the detection of foodborne pathogens in recent years due to their intriguing optical, electrical, and catalytic properties. The most attractive candidates for designing rapid and sensitive biosensors are these materials because of their large surface area, localized surface plasmon resonance, and impressive biocompatibility (Kapoor et al., 2024). The most recent advancements in MNP-based biosensing platforms can detect foodborne pathogens at ultra-low concentrations, even in the complexity of food matrices, such as *Escherichia coli, Salmonella enterica,* and *Listeria monocytogenes.* Stability, scalability, and toxicity issues need to be addressed to make possible significant applications in the food industry. Such amounts of interest are attracted concerning potential applications of gold nanoparticles as well as optical properties involved regarding colorimetric and electrochemical biosensing technologies. The latest application studies report that gold nanoparticles become especially significant for real-time applications (Awlqadr et al., 2024). For instance, recently, Magar et al. (2024) have developed a disposable paper-based biosensor that used Au nanoparticles conjugated with antibodies for the identification of *E. coli* O157:H7 in food samples with an LOD of 5 CFU/mL in a time of less than 20 minutes (Magar et al., 2024). Similarly, in developing an electrochemical biosensor, functionalized nucleic acids along with AuNPs were used for detecting very femtomolar concentrations of *Salmonella enterica* by Awang et al. (2021). Thus, the improvement of AuNPs toward specificity and sensitivity within the detection device strongly suggests a highly achievable property for PoC diagnosis (Awang et al., 2021). Another very popular group of nanoparticles with a well-known strong antimicrobial effect is AgNPs. AgNPs have also been reported to exhibit SERS properties (Zhang, et al., 2016). A lateral flow assay based on Surface-enhanced Raman spectroscopy (SERS) by using gold nanoparticles was fabricated for the rapid detection of *Listeria monocytogenes* in milk. The system achieved a limit of detection (LOD) of 75 CFU/mL under optimized conditions with remarkable practicality within food matrices (Wu, 2019). Recently, fluorescence-based biosensors incorporating AgNPs were designed for targeted recognition of *Salmonella* and *E. coli* in meat food and juice matrices and showed higher sensitivities and specificities—even in complex environments. Silver NPs exhibit superior optical and antimicrobial properties and are thus highly competent in applications where their detection/preservation is required (Zhang, et al., 2022).

Copper nanoparticles (CuNPs) and copper metal-organic frameworks (Cu-MOFs) have also gained attention as a cost-effective alternative to gold and silver nanoparticles, which provide comparable catalytic and optical properties for the detection of foodborne pathogens (Chen et al., 2021). Perk et al. (2023) achieved the development of a biosensor integrating copper metal-organic frameworks with gold nanoparticles for the detection of *Salmonella* and *Vibrio cholerae* in seafood at 0.5 CFU/mL. This study further demonstrated the application of copper-based nanomaterials in the identification of pathogens, which was relevant in resource-scarce environments (Perk et al., 2024). Gold-silver and copper-silver bimetallic nanoparticles also had synergistic interactions, which enhanced the detection sensitivity (Yu, Yang, et al., 2024). Redondo-Solano (2024) performed the application of gold-silver bimetallic nanoparticles for point-of-care diagnostics for the detection of *Escherichia coli, Listeria monocytogenes,* and *Salmonella* in 15 min (Redondo-Solano, 2024).

However, promising such potential may be, there are some challenges presented by the application of these metal-based nanoparticles in a real-life foodborne detection of pathogens. It will be about the stability of complex food matrices that often include pH, ionic strength, and organic compound presence that might interfere with their functionality (Bajpai et al., 2018) (Burlec et al., 2023). To overcome these challenges, efforts in the development of eco-friendly and biocompatible coatings, including polymeric or protein-based shells, to stabilize nanoparticles and reduce toxicity, are needed. The synthesis and functionalization of nanoparticles are being advanced to allow for the development of sophisticated biosensors. Thus, surface modification techniques, by adding biorecognition components such as antibodies, aptamers, and peptides, have significantly improved the specificity and reusability of nanoparticle-based sensors. Integration with metal-based nanoparticles, lab-on-chip systems, and portable devices is expected to soon revolutionize point-of-care diagnostic (POC) applications (Madhumathi et al., 2010; Popescu & Ungureanu, 2023). Mohanta and Chakraborty (2024) noted that recent efforts have indicated the possibility of using bimetallic nanoparticles for pathogen detection through lab-on-chip platforms with IoT-based monitoring protocols. Another increasing trend is the use of plant extracts and microbial-based synthetic approaches. This approach not only green-synthesizes but also leads to the possibility of eco-friendly nanosynthesis (Kyaw et al., 2024). Therefore, adopting green synthesis techniques using plant or microbial routes not only improves biocompatibility but also aligns with environmental safety and regulatory compliance, making them ideal for future food safety platforms. These prospects could be achieved through interdisciplinary research on nanotechnology, material science, and food microbiology to overcome the challenges identified by developing such biosensors. Further improvement in the stability, biocompatibility, and scalability of metal-based nanoparticles will allow them to realize their full potential in ensuring food safety and public health.

### 2.2 Carbon-based nanomaterials

The increasing prevalence of foodborne illnesses necessitates the development of rapid, sensitive, and reliable pathogen detection platforms. The landscape of biosensing technology has changed with the advent of carbon nanomaterials, such as graphene, carbon nanotubes (CNTs), graphene quantum dots (GQDs), and carbon dots (CDs). Such outstanding properties of these materials, encompassing high electrical conductivity, large surface area, and ease of functionalization, have been applied to electrochemical and optical sensing with great performance metrics. For example, electrochemical biosensors based on graphene can achieve a very low detection limit down to 0.1 CFU/mL for *Salmonella* (Muniandy et al., 2019a, Muniandy et al., 2019b), while CNT-based sensors have achieved a detection limit of 102 CFU/mL with a response time of fewer than 5 minutes for *E. coli* (Yamada et al., 2014). Similarly, carbon dots functionalized with biorecognition molecules enable fluorescence-based optical biosensors with detection limits of 10 CFU/mL in food matrices (Zheng et al., 2023). Despite these advancements, some severe limitations persist with the Carbon nanomaterial (CNM) CNM-based detection platforms. Reproducibility continues to be a major challenge in fatty, protein-rich, or fibrous foods with complex matrices, wherein nonspecific interactions and matrix effects undermine sensor accuracy. Another big challenge is that the synthesis process of CNMs usually involves complex and costly steps, which confines their scalability and practical applications. This problem was further compounded by the lack of standardization in synthesis protocols, which led to inconsistent sensor performance from one study to another (Bobrinetskiy et al., 2021). Moreover, although CNM-based biosensors exhibited outstanding performance in controlled laboratory settings, most of them suffered from lower sensitivity and specificity when applied to mixed microbial populations or real-world food samples. For instance, a SWCNT-based biosensor showed a linear detection range of 10^2^–10^5^ CFU/mL for E. coli, but matrix interference decreased its accuracy in milk samples (Yamada et al., 2016). Mechanistically, CNMs enhanced the performance of biosensors by facilitating the immobilization of biorecognition elements and efficient electron transfer. The large surface area of graphene enabled high-density functionalization with aptamers, antibodies, or enzymes, and its conductivity amplified the electrochemical signals. Quantum confinement effects in carbon dots were exploited for fluorescence-based detection by optical biosensors with high sensitivity (Zhang et al., 2020a, Zhang et al., 2020b). Carbon dots with magnetic nanoparticles, in addition to improving detection efficiency and specificity, are also exemplified by an electrochemical biosensor that can detect *E. coli* with a detection limit of 6.88 CFU/mL in milk and water samples (Lin et al., 2021). Overcoming current barriers, future research should focus on the cost-effective and scalable synthesis method - perhaps green chemistry approach-related development. Standardizing CNM preparation protocols as well as functionalization would possibly be one of the measures toward better reproducibility and reliability across different applications (Bobrinetskiy et al., 2021). Addressing challenges in complex food matrices required advanced surface engineering techniques, such as polymer coatings or hybrid composites, to minimize nonspecific interactions and matrix effects (Zheng et al., 2023a, Zheng et al., 2023b, Zheng et al., 2023c). Integrating CNM-based sensors with digital technologies, such as artificial intelligence and the Internet of Things (IoT), can enable real-time monitoring and predictive analytics for food safety applications (Temilade Abass et al., 2024). In summary, the development of multiplex biosensors capable of detecting several pathogens will greatly enhance the functionality and efficiency of detection systems for foodborne pathogens. Indeed, carbon nanomaterials have enormous potential to revolutionize foodborne pathogen detection because they exhibit outstanding sensitivity and specificity as well as fast response rates. Their existing limitations can be mitigated through innovative engineering and the introduction of novel technologies, which would unfold their full potential and provide a safer and more dependable food system worldwide.

One of the promising approaches for overcoming these limitations was carbon nanomaterial integration with hybrid systems, such as graphene-metallic nanoparticles or graphene-biopolymers. Hybrid biosensors using functionalized gold nanoparticles with graphene oxide can detect various pathogens simultaneously and thus overcome the limitations of the platforms designed to detect a single pathogen with higher specificity. These enable the development of CNM biosensors for rapid and low-cost foodborne pathogens detection in resource-poor settings. Additional portable smartphone-integrated point-of-care diagnostic technologies will aid in this regard (Solanki et al., 2022). The application of CNM-based sensors in practical applications is highly dependent on their ability to mitigate the effects of matrix interference. Polymer-coated CNMs or magnetic nanocomposites have shown promise in reducing nonspecific binding and enhancing the detection sensitivity in complicated food matrices such as milk, meat, and produce. For example, an electrochemical biosensor based on magnetic nanoparticles demonstrated a recovery rate of 94.7–103.8 % for *Listeria monocytogenes* from lettuce samples; such strategies were considered viable (Wang, et al., 2017). Quantitative improvement in detection performance always underlines the supremacy of CNMs-based biosensors. For instance, carbon nanobeads that integrate fluorescent carbon nanobeads into lateral flow assays are capable of reaching detection limits down to 10^2^ CFU/mL for *Staphylococcus aureus* and even 0.01 ng/mL for aflatoxin within food samples, therefore showing versatility and sensitivity (Deng et al., 2022). Furthermore, advances in impedimetric biosensors utilizing CNTs and gold nanoparticles have enabled the detection of *Salmonella enteritidis* at 10^4^ CFU/mL in milk, significantly improving response time to under three minutes (Kim et al., 2007). Shortly, combining CNMs with microfluidic devices and algorithms from machine learning will form a major platform for the development of the next generation of pathogen detection platforms. Thus, real-time, automatically analyzed food-borne pathogen analysis to unprecedented resolution and scale becomes possible. Moreover, sustainability is guaranteed through the integration of renewable and biodegradable materials during the synthesis of the CNMs, thereby significantly increasing the commercial potential for these technologies. Continuing innovations and interdisciplinary collaboration will eventually transform the food safety monitoring landscape by bringing CNM-based biosensors into play, thus reducing the global burden of foodborne illnesses and improving public health outcomes.

### 2.3 Polymeric nanoparticles

There has been an increased interest lately in the development of polymeric nanoparticles for the detection of foodborne pathogens because they provide unique properties such as biocompatibility and tunable surface properties that suppress interference from food matrices. They can encapsulate, hence enabling targeted recognition of pathogens as well as amplifying signals mitigating some of the critical challenges posed by the food matrix, including complex chemical compositions, interferents, and low amounts of pathogens. Polymeric nanoparticle-based systems present better sensitivity, portability, and appropriateness for real-time monitoring compared to conventional techniques, which include culture-based assays and PCR.

New trends indicated biosensors with polymeric nanoparticles that can promptly detect foodborne pathogens. A review of the microfluidic biosensor integrating the incorporation of polymeric nanomaterials for pathogenic detection, including *E. coli, Listeria monocytogenes,* and *Salmonella,* was covered by Weng et al. (2021). Polymeric nanoparticles were incorporated into the microfluidic system and yielded shortened assay time with an improved sensitivity even in complex food matrices. This was made possible by the efficient sample flow and accurate capture of pathogens (Weng et al., 2021). These systems also minimize the sample volume requirement and are thus suitable for high throughput analysis. Surface-modified polymeric nanoparticles on electrochemical biosensors are sensitive with minimal matrix effect. Dendrimers and conductive polymers as electrochemical nanocomposites in foodborne pathogens detection have been shown by Wang et al. (2023). These nanocomposites were included in the system, leading to enhanced efficiency of electron transfer and increased signal-to-noise ratio, so that pathogens like *E. coli* and *Salmonella* could be detected at concentrations of 10-100 CFU/mL (Wang et al., 2023). Another interesting point is that polymeric nanoparticles have been used very effectively for on-site colorimetric biosensors. Zhang, Ren, and Chingin (2020) emphasized the potential of functionalized nanoparticles of polydopamine in the design of colorimetric assays that detect *Salmonella* enteritidis in food products after processing. Using specific aptamers attached to polymeric nanoparticles, pathogens could be identified visually without the need for more sophisticated instrumentation. This system was able to achieve detection limits as low as 10 CFU/mL and showed greater resilience to matrix interference (Zhang et al., 2020). The past couple of years have been so momentous with the use of magnetic polymeric nanoparticles in pathogen isolation and detection in complex food matrices. Xiao et al. (2022) designed polymeric magnetic nanocarriers that were functionalized with receptors for effective separation and concentration of foodborne pathogens such as *Listeria monocytogenes* and *E. coli* from dairy and meat samples. It was discovered that the magnetic polymeric nanoparticles possess a particular pathogen capture ability at low background interference, and thus a high sensitivity of detection with a threshold of less than 10 CFU/mL (Xiao et al., 2022). Similarly, polymeric nanoparticles also have been used in DNA aptasensors to detect pathogens. Wu et al. (2020) demonstrated the use of polymer-coated gold nanoparticle-aptamer conjugates for the detection of *Listeria monocytogenes.* The conjugation of the polymeric nanoparticles with the aptamers made the detection of pathogens sensitive and specific with a detection limit of 1 CFU/mL in contaminated food samples (Wu et al., 2020). They are significant for enhanced foodborne pathogen detection because they show high specificity, lack susceptibility to interference from matrices, and result in the amplification of signals. Functionalization with biorecognition parts such as antibodies and aptamers made it feasible to produce highly sensitive biosensors amenable to onsite and real-time assessment. Accordingly, efforts in the future should go towards improving the scalability and reducing the cost, which will surely gain assurance of regulatory approvals for the applications of these polymeric nanoparticle-based systems to extend wide into food safety surveillance systems.

### 2.4 Hybrid nanomaterials

It represents a new class of detection agents for foodborne pathogens. This is because two or more nanomaterials can combine to offer synergistic properties, which include stability, conductivity, and multifunctionality. Therefore, the hybrid system offers better sensitivity, selectivity, and versatility relative to the individual nanomaterial advantage that can be exploited in their applications in biosensing and safety surveillance systems (Godja & Munteanu, 2024). Some of the breakthroughs allow for the in situ fast detection of pathogen agents such as *Salmonella enterica, Escherichia coli,* and *Listeria monocytogenes.* Among hybrid nanomaterials, the most recent remarkable innovation breakthroughs include metal-carbon hybrids. Hybrid nanomaterial-based carbon allows the creation of detection functionalities based on intrinsic optical properties of metals with electrical conductivity (Sheikhzadeh et al., 2021). For example, Mei et al., 2022a, Mei et al., 2022b developed an electrochemical biosensor with gold nanoparticle-graphene hybrids to detect *Salmonella* at a limit of 1.2 CFU/mL (Mei et al., 2022). In a related study, Kumar et al. (2020) showed the synthesis of hybrid nanomaterials comprising gold nanoparticles and quantum dots, where optical detection is coupled with antimicrobial activity against food-related pathogens. Also, gaining much importance are polymer-metal hybrids that are biocompatible and exhibit superior functional properties. Hybrid nanocomposite sensor based on chitosan, graphene oxide, and iron oxide for the electrochemical sensing of *E. coli*. It exhibited very fast response times coupled with detection thresholds of 0.3 CFU/mL in milk samples (Kumar et al., 2020). Ullah et al. (2024) synthesized polymer-coated silver nanoparticles incorporated with carbon nanotubes, showing remarkable stability and sensitivity toward the identification of foodborne pathogens. Hybrid nanomaterials that integrate magnetic properties have enabled the construction of systems that can be utilized for the effective separation and detection of pathogens (Ullah et al., 2024). Manoswini, Majumdar, Pany, Sahu and Mohanty, 2023a, Manoswini, Majumdar, Pany, Sahu and Mohanty, 2023b developed a hybrid biosensor made of magnetic nanoparticles and graphene oxide for the detection of *Listeria monocytogenes.* This system reached 0.8 CFU/mL with sample preparation through the technique of magnetic separation (Manoswini et al., 2023). Bobrinetskiy et al. synthesized in the year 2021, a hybrid magnetic nanoparticle system designed for the detection of concurrent multiple pathogens; it can be used with multiple food testing, among other optical hybrids including plasmonic nanoparticles hybrids and fluorescent dye (Bobrinetskiy et al., 2021). A new synthesis of a carbon nanotube and silver nanoparticle-based surface-enhanced Raman scattering sensor is presented to achieve rapid, high-sensitive, and excellent signal-to-noise ratio *Listeria* detection by Zhang et al. in 2022 (Zhang, Zhang, et al., 2022).

**Fig. 2:**
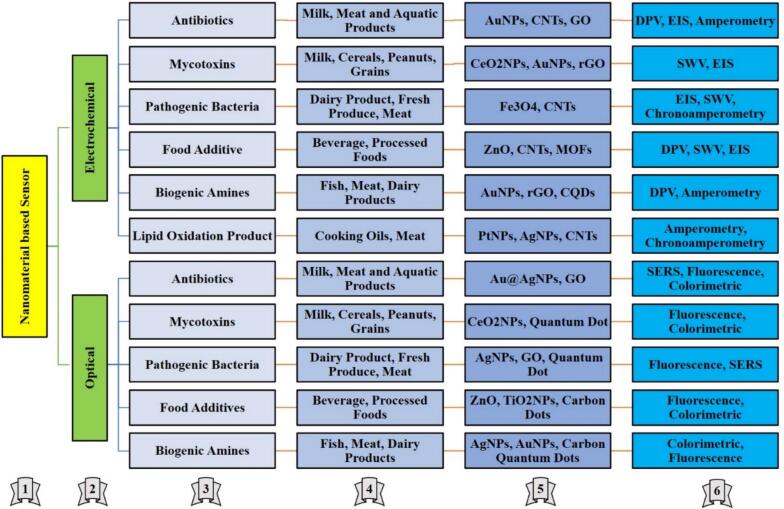
Overview of Nanoparticle-Based Sensors for Food Safety Applications. The figure is structured as follows: **(1)** Nanoparticle-Based Sensor (main title), **(2)** Sensor Type (Electrochemical or Optical), **(3)** Analyte (e.g., Antibiotics, Mycotoxins, Pathogenic Bacteria, Food Additives, Biogenic Amines, Lipid Oxidation Products), **(4)** Sample (e.g., Milk, Meat, Aquatic Products, Cereals, Beverages, Cooking Oils), **(5)** Nanomaterial (e.g., AuNPs: Gold Nanoparticles, AgNPs: Silver Nanoparticles, CNTs: Carbon Nanotubes, rGO: Reduced Graphene Oxide, CeO2NPs: Cerium Oxide Nanoparticles, MOFs: Metal-Organic Frameworks), and **(6)** Technique Used (Electrochemical Techniques: DPV - Differential Pulse Voltammetry, EIS - Electrochemical Impedance Spectroscopy, SWV - Square Wave Voltammetry; Optical Techniques: SERS - Surface-Enhanced Raman Spectroscopy, Fluorescence, Colorimetric)

Despite their great potential, hybrid nanomaterials present many challenges in practical applications. One of them is stability in complex food matrices, where pH, ionic strength, and organic compounds can interfere with the sensor performance. Valenzuela-Amaro et al. (2023) dealt with this by introducing biopolymer coatings to stabilize hybrid nanomaterials and prevent agglomeration. Another challenge is the scalability of fabrication methods because hybrid materials often require complex synthesis procedures (Valenzuela-Amaro et al., 2023a). In 2023, Zhao et al. noted the need for cost-effective techniques that can be reproducibly scaled up for widespread production. Hybrid nanomaterials, especially those containing metallic components, are of concern for their environmental and biological impacts (Zhao et al., 2023). Du et al. in 2022 suggested the use of biocompatible coatings and green synthesis. The integration of hybrid nanomaterials into portable diagnostic devices and IoT-based systems remains challenging (Du et al., 2022). According to Zheng et al. (2023), advanced sensor designs were required to create field-deployable, real-time monitoring solutions (Zheng et al., 2023). Hybrid nanomaterials can revolutionize foodborne pathogen detection by combining the best features of different materials. Innovations such as multifunctional hybrids, green synthesis approaches, and lab-on-chip integration are likely to address current challenges and unlock new possibilities for ensuring food safety. Further Table 1 summarises key advancements, applications, detection limits, material scalability, safety profiles, and future research directions of nanoparticle-based biosensors for pathogen detection. Figure 2 shows an overview of nanoparticle-based sensors for food safety applications. The figure is structured as follows: (1) nanoparticle-based sensor (main title), (2) sensor type (electrochemical or optical), (3) analyte (e.g., antibiotics, mycotoxins, pathogenic bacteria, food additives, biogenic amines, lipid oxidation products), (4) sample (e.g., milk, meat, aquatic products, cereals, beverages, cooking oils), (5) nanomaterial (e.g., AuNps: gold nanoparticles, AgNps: silver nanoparticles, CNTs: carbon nanotubes, GO: Graphene oxide, rGo: reduced graphene oxide, Ceo2Nps: cerium oxide nanoparticles, MOFs: metal-organic frameworks), and (6) technique used (electrochemical techniques: DPV - differential pulse voltammetry, EIS - electrochemical impedance spectroscopy, SWV - square wave voltammetry; optical techniques: SERS - surface-enhanced Raman spectroscopy, fluorescence, colorimetric).

**Table 1:** Summary of key advancements, applications, detection limits, material scalability, safety profiles, and future research directions of nanoparticle-based biosensors for pathogen detection

Nanoparticle Type	Key Advancements	Applications	Detection Limits and Time	Material Cost and Scalability	Biocompatibility/ Safety	Representative Pathogens Detected	Future Research Focus Areas	References
**Metal-Based Nanoparticles**	High sensitivity due to plasmonic and catalytic properties; rapid detection; potential for multiplexing with bimetallic systems.	Colorimetric, electrochemical, and SERS-based biosensors for detecting *Salmonella*, *E. coli*, and *Listeria*.	1–10 CFU/mL within 20–30 minutes (varies by sensor type).	Moderate to high cost (gold/silver); synthesis scalable but dependent on material purity.	Potential toxicity of metals (silver, copper); requires biocompatible coatings.	*Salmonella*, *E. coli*, *Listeria monocytogenes*, and *Vibrio cholerae*.	Eco-friendly synthesis; development of portable multiplex sensors; improved biocompatibility.	(Manoswini et al., 2023b; Mei et al., 2022b; Pathania et al., 2023)
**Carbon-Based Nanomaterials**	Exceptional electrical conductivity; high surface area; fluorescence and Raman-based enhanced detection systems.	Electrochemical and fluorescence-based sensors for pathogens like *Salmonella* and *E. coli*; carbon dots for low-concentration detection.	As low as 0.5 CFU/mL; rapid response under 15 minutes (fluorescence-based sensors).	Moderate cost; challenging scalability due to limited synthesis techniques.	Biocompatible but stability challenges under food matrix conditions.	*Salmonella*, *E. coli*, and noroviruses.	Scalable green synthesis; enhanced stability in food matrices; IoT-based integration.	(Bobrinetskiy et al., 2021; Fatemi et al., 2024; Muniandy et al., 2019b)
**Polymeric Nanoparticles**	Customizability; stimuli-responsive designs; molecular imprinting for selectivity; multifunctional biosensors.	Electrochemical biosensors for *Listeria* and *E. coli*; antimicrobial food packaging with detection capabilities.	1–5 CFU/mL detection with variable time depending on stimuli-responsiveness.	Low to moderate cost; scalable using green synthesis approaches for biopolymers.	High biocompatibility; toxicity possible with certain additives (e.g., metals).	*Listeria monocytogenes*, *E. coli*, and *Salmonella*.	Cost-effective hybrid materials; stimuli-responsive systems for on-site diagnostics; regulatory advancements.	(Park et al., 2024; Pathania et al., 2023; Xu et al., 2016)
**Hybrid Nanomaterials**	Synergistic properties; combining strengths of multiple materials for improved stability, sensitivity, and multifunctionality.	Multiplex detection platforms; enhanced signal amplification; magnetic separation for complex food matrices.	0.3–0.8 CFU/mL with efficient multiplexed detection; response times under 15 minutes.	High cost; scalability limited by complex fabrication and need for hybrid precision.	Variable; depends on components (metal-carbon hybrids require careful design to reduce toxicity).	*Listeria*, *E. coli*, *Salmonella*, and multiple pathogens in multiplex systems.	Simplified hybrid synthesis; green fabrication; and enhanced regulatory frameworks for multi-functional systems.	(Ungureanu et al., 2022; Valenzuela-Amaro et al., 2023b; Zheng et al., 2023c)

## 3 Comparative analysis of detection techniques

Foodborne pathogens pose significant threats to public health and food safety worldwide. To mitigate these risks, the field of pathogen detection has progressed from conventional microbiological methods to advanced molecular and nanotechnology-based systems. This section provides a critical analysis of these detection techniques, emphasizing their evolution, current capabilities, limitations, and prospects (as shown in Table 2). Culture-based methods are still the cornerstone of pathogen detection because they are reliable and can confirm viable organisms. They imply isolation and identification of the pathogens on selective media. Biochemical confirmation then takes place. Although very precise, they were slow and very labor-intensive, and in most cases, the outcome takes 24–72 hours. This becomes their major drawback for use in time-sensitive applications like outbreak investigations or real-time quality control (Todd, 2020). Advances in techniques, including polymerase chain reaction, and related molecular techniques significantly shortened detection times while improving sensitivity and specificity. PCR, quantitative PCR, and loop-mediated isothermal amplification make it possible to amplify pathogen-specific genes within a detection limit of less than 10 copies in a little more than an hour (Liu et al., 2021). However, the methods were expensive, needing skilled personnel and large amounts of preparation of samples; therefore, not the most appropriate for field use or resource-limited applications. CRISPR-Cas systems have dramatically altered the detection of pathogens in their high specificity and sensitivity, ease of use, and programmability. Such systems employ programmable nucleases, Cas12a and Cas13, to recognize and cleave pathogen-specific nucleic acids. Example. Biosensors based on the CRISPR-Cas12a system were applied for fast and accurate diagnosis of Salmonella within an ultra-sensitivity limit of 1 CFU/mL in one hour (Ma et al., 2021). Hybrid methods have increased the CRISPR system amplification. RPA-Cas12a-FS is a combination of recombinase polymerase amplification with the CRISPR system that will enable the detection of 10 DNA copies in concentration for 45 minutes under isothermal conditions. The system, therefore, would be appropriate for decentralized food safety monitoring (Zhou et al., 2024). Furthermore, the association of CRISPR-Cas12a with SERS has shown detection limits in the femtomolar range, thus even further extending the boundaries of what is possible in terms of sensitivity (Teng et al., 2024). Nonetheless, CRISPR systems still have several problems. Nucleic acid amplification steps complicate workflows and may introduce errors, and the need for additional optimization to achieve true multiplexing remains a significant problem (Yu, Huang, et al., 2023). Nevertheless, the versatility and adaptability of CRISPR-based diagnostics promise transformative applications in food safety.

**Table 2:** Enhanced comparison of detection techniques for foodborne pathogens.

Parameter	Traditional Methods	Emerging Technologies	Nanoparticle-Based Systems
**Time-to-Result**	24-72 hours *Example*: Listeria detection during outbreaks	1-6 hours *Example*: CRISPR-based *Salmonella* detection	Minutes to hours *Example*: *E. coli* biosensor using gold nanoparticles
**Sensitivity**	∼10^2 CFU/mL Limited for low pathogen concentrations	∼10 CFU/mL Effective for specific pathogens	<1 CFU/mL Ultra-sensitive even in trace amounts
**Specificity**	Moderate Relies on general detection protocols	High Enabled by CRISPR precision	Ultra-high Enhanced by functionalized nanoparticles
**Portability**	Limited Lab-bound techniques requiring large setups	Moderate Miniaturization improves accessibility	Portable Field-deployable systems available
**Scalability**	High Well-established for industrial scale	Moderate Scalability under development	Moderate Adaptable for small-scale industrial use
**Cost**	Low Inexpensive due to simple tools and reagents	Moderate to High Requires advanced materials	Moderate to High Costs depend on nanoparticle synthesis.
**Operational Complexity**	High Requires skilled personnel and lab equipment	Moderate Simpler than traditional methods but still needs expertise	Low User-friendly biosensors designed for field use
**Interference Robustness**	Low Struggles with complex food matrices like dairy or seafood	Moderate Better suited for liquid matrices	High Engineered nanoparticles resist matrix interference.
**Reference**	(Chen et al., 2017; Kabiraz et al., 2023; Petrucci et al., 2021; Zhou et al., 2017)	(An et al., 2021; Bhowmik et al., 2024; “Emerging non-thermal technologies for decontamination of Salmonella in food | Request PDF,” 2024; Gao et al., 2022; Kaavya et al., 2021)	(Ghazy et al., 2024; Guruprasath et al., 2024; Hegde et al., 2022; Sadanandan et al., 2023; Wang & Alocilja, 2015)

**Fig. 3:**
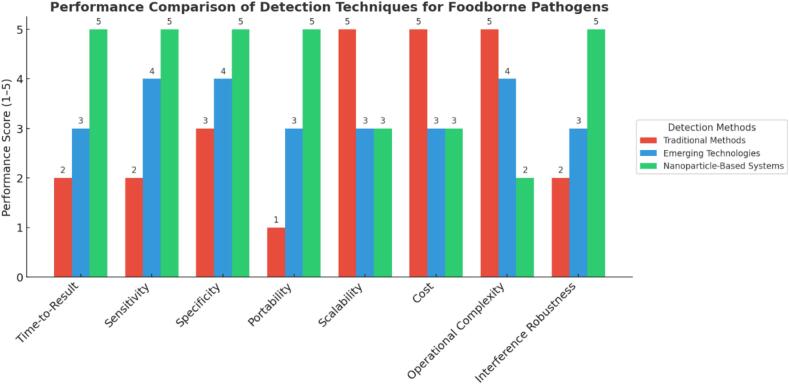
Performance Comparison of Foodborne Pathogen Detection Techniques. This bar graph compares the performance of three detection methods—Traditional Methods, Emerging Technologies, and Nanoparticle-Based Systems—across eight key parameters: Time-to-Result, Sensitivity, Specificity, Portability, Scalability, Cost, Operational Complexity, and Interference Robustness. Scores are normalized on a 1-5 scale, with 5 indicating the highest performance.

Nanotechnology has emerged as an excellent tool for the creation of highly sensitive and fast detection of foodborne pathogens. Among all kinds of nanoparticles, gold nanoparticle is widely used because of its unique optical property in colorimetric detection. Recently, gold-nanoparticle-based biosensors were developed to detect *Salmonella,* achieving the limit of detection of 10 CFU/mL in under 8 hours (Jiang et al., 2023). More recent studies have expanded the potential for the detection of foodborne pathogens using upconversion nanoparticles, which have unique fluorescence properties. UCNP-based arrays were able to detect mixtures of bacteria in real food samples with an accuracy of more than 92%, showing the high-throughput and multiplexed analysis potential (Abdul Hakeem et al., 2022). Integration of nanoparticles with CRISPR systems has opened up new avenues for ultra-sensitive and rapid detection. For example, hybrid systems consisting of CRISPR-Cas12a-nanoparticles have reached sub-femtomolar detection limits, which combine the programmability of CRISPR with the optical and catalytic properties of nanoparticles (Suliman Maashi, 2024). However, for these nanomaterials to gain widespread acceptance, their scalability, reproducibility, and environmental impacts have to be addressed. Integration of CRISPR systems with nanotechnology represents the future of pathogen detection. Combining molecular precision in CRISPR with the sensitivity and versatility of nanoparticles would allow researchers to design very portable, multiplexed platforms that could be monitored in real-time within a variety of environments. In terms of cost-benefit, conventional culture-based methods are typically inexpensive in materials but incur high labor and time costs, making them inefficient for rapid outbreak management or large-scale food processing environments (Alamer et al., 2017). PCR and CRISPR systems, while offering faster and more sensitive detection, require skilled personnel, complex workflows, and costly reagents, limiting their accessibility in low-resource settings (Peterson et al., 2022). In contrast, nanoparticle-based platforms offer a favorable cost-benefit ratio, especially when scaled up. Once developed, these systems can be miniaturized into portable, low-cost sensors requiring minimal training and rapid results, making them ideal for routine screening in food industries (Jiang et al., 2023), (Song et al., 2024). Though initial R&D and material synthesis costs are high, nanoparticle systems offer long-term affordability, particularly with scalable production and integration into user-friendly formats. Future research work must focus on technological advancement for the facilitation of simplification in workflow, multiplexing, and cost-effectiveness for generalization. The detection technology of pathogens has evolved from conventional to labor-intensive and time-consuming to modern molecular biology and nanotechnology-based techniques. Conventional techniques will be very essential in confirmatory testing, but CRISPR-based systems and nanoparticle-enabled platforms constitute breakthrough approaches concerning speed, sensitivity, and scalability. To make full capabilities in food safety diagnostics a reality, it will be important to reduce existing constraints and encourage cross-disciplinary cooperation. Lastly, the bar graph (Figure 3) compares the performance of three detection methods—Traditional Methods, Emerging Technologies, and Nanoparticle-Based Systems—across eight key parameters: Time-to-Result, Sensitivity, Specificity, Portability, Scalability, Cost, Operational Complexity, and Interference Robustness. Scores are normalized on a 1-5 scale, with 5 indicating the highest performance.

## 4 Food matrix-specific challenges

**Fig. 4:**
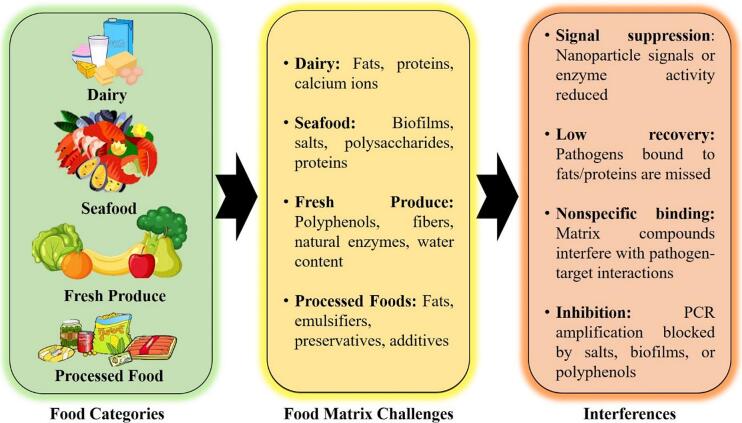
Food categories, associated matrix challenges, and key interferences impacting nanoparticle-based sensor performance in food safety detection.

Detecting pathogens in complex food matrices is a significant challenge because of the diverse chemical and physical properties of different food types. These matrices, ranging from dairy products to seafood and fresh produce, often contain components such as fats, proteins, salts, and biofilms that interfere with the performance of the detection systems (as shown in Figure 4). To address these obstacles, tailored nanoparticle-based solutions are required to ensure accuracy, sensitivity, and robustness.

### 4.1 Dairy products

Dairy products such as milk, cheese, and yogurt are composed of nutrient-rich ingredients, so they are prone to microbial contamination. The key to ensuring food safety is pathogen identification. However, the complex constituents of the dairy matrix add a layer of complexity, making it challenging for detection systems, especially those based on nanoparticles. Difficulties occur because dairy products have specific physicochemical properties, including high-fat content, variability in protein composition, and non-uniform processing procedures, making it difficult to detect qualitative specificity and reliability. Hydrophobic conditions triggered by high-fat contents within the dairy matrices destabilize the nanoparticles, causing them to aggregate and leading to the loss of signal integrity. This is a particularly difficult challenge in systems that utilize SPR or fluorescent detection because nanoparticles that lack stability negate the consistency of the signal. Milk fat globules can reduce the sensitivity of gold nanoparticle systems by up to 40% (Li et al., 2020). Besides, casein and whey proteins interfere with the operational functionality of nanoparticles through their adhesion to the nanoparticle surface, causing a nonspecific interaction that shields active sites, thereby creating reduced specificity. The ordinary aptamer-functionalized gold nanoparticles showed a 30% decrease in detection efficacy in untreated milk, implying protein interference effects (Hendrickson et al., 2024). Furthermore, differences can be explained in dairy matrices through differences in methods such as pasteurization, homogenization, and other fermentation processes used. Such diversity requires responsive and dependable detection systems that would work well in various types of dairy products (Sadeq et al., 2024).

Recent advances in nanotechnology technology have provided fresh solutions for these problems. Surface modification, including adding PEG or silica-based coatings to the nanoparticles increases the nanoparticle stability tremendously in the fat-rich milieu. Nanoparticles attached with PEG maintained detection efficiency as high as 95% in milk than their naked counterparts in regard to both stability and sensitivity (Kalińska et al., 2024). Hybrid systems consisting of magnetic nanoparticles with biosensors combine the advantages of specificity and reduced matrix interference. The hybrid systems have been demonstrated to detect Staphylococcus aureus in raw milk with detection limits as low as 1 CFU/mL, indicating potential for practical use (Gao et al., 2024). Recent advances in ultra-low concentrations that are possible by using pretreatment processes, such as ultrafiltration and enzymatic hydrolysis, in lowering the fat and protein interferences improve the sensitivity to nanoscale particle-based detection of *Salmonella typhimurium*. The pretreatment methodology allows processed cheese to be analyzed with a 3-log improvement in sensitivity (Zhu et al., 2024). Photoelectrochemical detection systems are yet another innovation in modalities. These rely on modes of dual signal, either fluorescence or electrochemiluminescence, thus allowing for specificity even with complex matrices. For example, silver nanoparticle-based platforms have been used for the detection of *E. coli* O157:H7 in homogenized milk with unprecedented accuracy (Chen et al., 2020). These are remarkable breakthroughs, but there is still more work that needs to be done before such technologies reach industrial scales. Economic barriers, like the high cost of synthesizing nanoparticles and regulatory barriers regarding the use of nanoparticles in food safety, have to be removed. Global standards harmonization and inexpensive production methods unlock the door for these technologies. The integration of nanoparticle-based detection systems into IoT devices and real-time monitoring platforms may revolutionize pathogen detection in dairy supply chains. These will enhance traceability, lead to quick response to contamination, and provide actionable data to stakeholders. To attain this vision, it has to be a multidisciplinary approach that brings material science advancements, engineering prowess, and regulatory policies under one roof.

### 4.2 Seafood: addressing challenges in pathogen detection

The complex structure of seafood is a particularly sophisticated food matrix that causes a number of analytical complexities. High protein, lipid, and ionic strength often result in matrix interferences responsible for signal suppression, nonspecific interaction, and loss of sensitivity with conventional detection techniques such as LC-MS or standard immunoassays (Wang et al., 2024). Such restrictions lead to long, expensive, and labor-intensive procedures. Nanoparticle-based technologies have been developed as revolutionary approaches to overcome food matrix challenges. These will offer sensitive, specific, and efficient detection of contaminants in seafood. For example, MNPs with biosensors have proved to be exceptional for detecting pathogens in food matrices. Peptide-probe-based paper magnetic nanoparticles are capable of rapidly detecting *E. coli* O157:H7 can be detected even down to a concentration as low as 30 CFU/mL in spiked seafood matrices. Such colorimetric biosensors offer a low-cost, portable approach to the monitoring process in real-time (Suaifan et al., 2017). UCNPs have actually revolutionized the detection strategy for chemical contaminants such as antibiotics and pesticides. The deep penetration of light and autofluorescence background give these nanoparticles an excellent scope for seafood matrices. Sensing based on UCNP has been developed for detection of enrofloxacin in fish samples with a minimum detection limit of 0.04 ng/mL with recovery rates up to 96% (Liu et al., 2017). Additionally, Rong et al. (2021) highlighted the potential of lanthanide-doped UCNPs for detecting heavy metals, pesticides, and antibiotics in seafood due to their stability and high sensitivity (Rong et al., 2021). Novel pollutants like nanoplastics have been found in seafood by using techniques modified by nanoparticles. Isolation and detection of 100 nm polystyrene nanoparticles in fish without the aggregation problem were achieved at a detection limit of 52 μg/g by Correia and Loeschner (2018) who used asymmetric flow field-flow fractionation (AF4) combined with multi-angle light scattering (MALS) (Correia & Loeschner, 2018).

Moreover, AuNPs have been exploited for the detection of markers and contaminants in seafood spoilage. Recently, Hua et al. (2021) emphasized the utility of AuNP-based biosensors for the biogenic amines and sulfide bioproducts, namely, two major indicators of seafood spoilage for fish and crustaceans. As the latter undergo a colorimetric reaction because of particle aggregation, their sensors may be detected by the naked eye and provide a direct, immediate evaluation of quality (Hua et al., 2021). Ultimately, the nanocomposites of quantum dots form versatile tools to identify contaminants. Xiong et al. developed magnetic fluorescent quantum dot nanocomposite, MNPs@QDs, incorporating magnetic separation techniques with fluorescent methods for detection in 2022. Such sensors allow for the concurrent identification of heavy metals, pathogens, and toxins in seafood matrices that ensure efficient pre-treatment of samples with amplified sensitivity (Xiong et al., 2022). These nanotechnology-based detection techniques find their importance due to superior sensitivity, specificity, and resistance to matrices over the conventional method. Inbuilt magnetic, optical, and up-conversion properties would help in overcoming some of the current limitations in detection methodology while making nanoparticle-based technology an efficient means to track pathogens, chemical residues, or emerging contaminants in any seafood at any point in time.

### 4.3 Fresh produce: challenges and advances in pathogen detection

The detection of foodborne pathogens in fresh produce**,** such as fruits and vegetables, presents considerable challenges due to the complex nature of the food matrix. Fresh produce contains high water content**,** natural enzymes**,** fibrous components**,** and bioactive compounds like polyphenols and organic acids, all of which can interfere with detection systems by reducing sensitivity, suppressing target signals, and hindering pathogen recovery (Bharti et al., 2024). Culture-based assays, PCR, and ELISA are relatively very good but possess some serious disadvantages in fresh produce matrices. For instance, culture-based techniques take up to 24–72 hours of the assay time in isolating the pathogens. The occurrence of PCR inhibitors that emanate due to matrix-associated interference faces challenges associated with rapid sensitivity in the detection of pathogens through the matrices of fresh produces. This can be related to contamination that can result from either pre-harvest or post-harvest handling. The current situation presents a revolutionary method to the detection technologies marked by high sensitivity, specificity, and the least interference with the matrix. Because of the advantages such as their optical properties superior compared with traditional semiconductor nanomaterials and signal-amplification capability, gold nanoparticles have found great versatility in pathogenic microorganism identification. Wang & Duncan, 2017 designed an oligonucleotide ligation-PCR combined microarray system by making use of gold nanoparticles to recognize eight kinds of typical pathogenic bacteria within fresh fruits and vegetables; those found pathogens were *E. coli* O157:H7*, Salmonella enterica, Listeria monocytogenes,* and many more. This system achieved great sensitivity, with detection limits ranging from as low as 3.3 CFU/mL for some pathogens up to rapid and cost-effective analysis (Wang, Ying, et al., 2017). Panchal et al. (2022) recently developed a plasmon-enhanced nanosensor that used gold and iron oxide nanozymes as enzyme mimetics to detect *E. coli* O157:H7. Replacing traditional enzymes like horseradish peroxidase (HRP) with enzyme catalysts, the system developed resulted in a detection time of less than 15 minutes, a 100-fold improvement in sensitivity compared to traditional ELISA methods (Panchal et al., 2022).

MNP-based systems were also very efficient for the isolation of pathogens from complex food matrices. Recently, a highly innovative FOSPR-based biosensor integrated Ag-NPs/GR-functionalized antimicrobial peptides were designed by Zhou et al., 2018). The minimum detectability with this system for the isolate *E. coli* O157:H7 was quantitated at 5 × 10^2^ CFU/mL in freshly squeezed juices from fruits and vegetables, and the recoveries ranged from 88% to 110%, so the sample could be directly analyzed for the presence of pathogens (Zhou et al., 2018). Nanoparticle-based sensors have presented promise for colorimetric biosensors that allow visual and field-ready detection. Zhang, Huang, et al. (2016) demonstrated a nanoparticle cluster-catalyzed biosensor for the visual and point-of-care detection of *Listeria monocytogenes* on fresh produce with a detection limit of 5.4 × 10^3^ CFU/mL and fast visual interpretation of the system comprising vancomycin-functionalized gold nanoparticles combined with Fe₃O₄ clusters for signal amplification (Zhang, Liu, et al., 2016). Fresh produce matrices have been said to reduce interferences through the use of the most up-to-date methodologies, including SERS, the work conducted by Wang et al. (2019), has reported foodborne pathogens and contaminants found in fruits like grapes utilizing the core-shell Au@Ag nanoparticles. The sensitivity of the limits of detection was very low, coupled with specificity, and no interference caused by inhibitors and matrice (Wang et al., 2019). These studies showed how nanoparticle-based detection technologies can mitigate the challenges of fresh produce matrices by providing rapid sensitivity and specificity in pathogen detection. Gold nanoparticles magnetic nanoparticles and SERS platforms offer enhanced signal amplification, reduced matrix interference, and direct applicability at the field level not achievable by traditional approaches; however, challenges remain concerning standardization of synthesis protocols for nanoparticles, scalability, and cost limitations for industrial applications. This integration of these technologies with portability devices and machine learning-based data analysis will further solidify them as more applicable methods for the real-time monitoring of foodborne pathogens in fresh produce, thereby ensuring food safety and public health.

### 4.4 Processed foods: overcoming challenges in pathogen detection with nanoparticle-based technologies

Processed food includes dairy products, pre-cooked food, preserved food, canned foods, and meats. Due to the presence of fats, proteins, salts, emulsifiers, preservatives, and additives, the identification of pathogens becomes difficult since it involves a food matrix. The reason for suppressing the signal and poor recoveries of pathogens could also be due to the interfering nature of these compounds, inhibitory in nature, associated with conventional techniques such as culture-based, PCR, and ELISA methods. Besides, pre-treatment of samples of processed foods sometimes proves to be too comprehensive in order to isolate the pathogens. This makes the test lengthy and complicated. In order to overcome the said limitations, nanoparticle-based detection technologies have been designed as effective tools for delivering enhanced sensitivity, specificity, and resistance to matrix interferences for the proper detection of pathogens in processed food matrices. MNPs have shown exemplary promise in the isolation and detection of pathogens from intricate processed food matrices. For example, Zou et al. (2019) developed a biofunctionalized magnetic nanoparticle cluster sensor coupled with NMR for the detection of Salmonella in spiked milk samples. By selectively binding pathogens using antibody-functionalized Fe₃O₄ nanoparticles, the system achieved a detection limit of 10^5^ CFU/mL and overcame signal suppression caused by high fat and protein content (Zou et al., 2019). Similarly, Zhao et al. (2017) reported a method using antibody-modified Fe₃O₄ magnetic nanoparticles to detect *Listeria monocytogenes* in milk powder and lettuce, achieving a sensitivity of 3 MPN (most probable number**)**. This NMR-based approach demonstrated high specificity and robustness against interfering matrix components (Zhao et al., 2017). QD-based immunofluorescence-based platforms have been considered in great detail within the realm of multiplex pathogen identification in processed food matrices. Wang et al. (2020) described that QD-functionalized immunomagnetic nanoparticle systems have been prepared for the concomitant detection of *E. coli* O157:H7, *S. aureus,* and *V. parahaemolyticus* in milk and other food matrices, and the system exhibited a detection limit of 6.6 × 10^0^ CFU/mL for *E. coli* and comparative sensitivities of other pathogens, within less than 4 hours. This technique was very well suited for pathogen monitoring in processed foods because of its high multiplexing capability and its resistance to matrix interference (Wang et al., 2020).

The quick screening of pathogens in processed food products is effectively possible with gold nanoparticles (AuNPs). Quintela and his colleagues designed an oligonucleotide-functionalized AuNP-based colorimetric biosensor for detecting *Salmonella spp*. from processed chicken and blueberries. It achieved very low detection limits below 10 CFU/mL due to the visible color changes through the aggregation of AuNPs, showing excellent specificity and sensitivity without extensive pre-treatment (Quintela et al., 2019). Similarly, Wu and his colleagues integrated the AuNP-based antibody-aptamer sandwich assays with enzyme-linked immunosorbent methods and achieved an efficient and selective detection of *Salmonella Typhimurium* in milk by a detection limit of 10^3^ CFU/mL (Wu et al., 2014). The nanozyme-mediated signal amplification-based dual lateral flow immunoassays have been highly promising in the real-time surveillance area. Cheng developed a smartphone-compatible LFIA, upgraded with Pd@Pt nanozymes, for the simultaneous detection of *Salmonella Enteritidis* and *E. coli* O157:H7 in deli meat products. The system attained detection limits of 20 CFU/mL and 34 CFU/mL within a time frame of 30 minutes. It offered a cost-effective and portable approach for in-field pathogen detection (Cheng et al., 2017).

To further reduce matrix-related interference, researchers have proposed advanced pre-treatment strategies. These include functionalized magnetic nanoparticles (MNPs) that enable targeted pathogen separation prior to detection. FPMNC-based immunoassays showed minimal matrix interference while capturing *Salmonella Typhimurium* with high specificity and without pre-enrichment, achieving detection limits as low as 138 CFU/mL in lettuce samples (Guo et al., 2020). Similarly, lectin-coated magnetic beads paired with Raman tags enabled interference-free SERS detection of multiple pathogens in food matrices with high sensitivity (Yang et al., 2022). For general food matrix challenges, culture-independent methods combined with preprocessing strategies such as selective lysis, centrifugation, and magnetic nanoparticle enrichment significantly reduce inhibitory effects and improve recovery of pathogens in complex samples (Jia et al., 2023), (Du et al., 2024). Another recent innovation involves magnetic fluorescent quantum dot nanocomposites, which integrate separation and detection in a single system, minimizing sample loss and interference (Xiong et al., 2022). These strategies offer a promising path to overcome food matrix limitations, improve analytical sensitivity, and bring nanoparticle-based biosensors closer to real-world deployment. To conclude, nanoparticle-based detection technologies, such as magnetic nanoparticles, quantum dots, gold nanoparticles, and enzyme-enhanced systems have indeed crossed food matrix barriers that go along with processed foods. It thus makes feasible rapid, sensitive, and multiplex pathogen detection without the interference that goes with fats, proteins, and additives within the matrix. More work on development, scale up, low-cost production, and wide industrialization of the area remain still. In contrast, the development in machine learning and use with suitable algorithms for portable devices shall propel the next generation pathogen detection within food processing matrix further. Hereby the safety shall go with conformity.

## 5 Integration with smart technologies: revolutionizing food safety monitoring

The integration of nanotechnology into smart systems is the new paradigm of food safety monitoring, where unprecedented data collection in real-time and predictive analysis would give way to supply chain transparency. Advanced nanosensors, AI, and blockchain technologies enable these systems to achieve more precise detection, reduced response times, and smooth operation, making them respond to the new demands of the food industry.

### 5.1 Real-time monitoring with embedded nanosensors

Real-time monitoring of food safety using nanosensor technology for real-time food safety monitoring, contamination prevention, and quality control is now performed in an extremely proactive manner (Sharma et al., 2023). Compared to conventional approaches involving periodic testing with retro data, nanosensors would enable the real-time tracking of continuous products within a given supply chain. Here, nanosensors have utilized nanoscale-specific properties such as their high surface-to-volume ratios and tunable surface chemistries, enabling sensitive detection of microbial contamination as well as shifts in conditions due to chemical spoilage indicators and changes in the environment. The most promising application of nanosensors is in smart packaging. They are embedded in food to monitor food quality at every point during storage and distribution (Chelliah et al., 2021). For example, gold nanoparticles may be used in the caps of milk bottles to detect spoilage gases, such as ammonia and hydrogen sulfide, resulting from the metabolic activity of bacteria. Such sensors detect in near-instant time frames and reach sensitivity thresholds of as low as 1 ppm (Yu, Zhao, & Xie, 2024). Silver nanoparticle-based colorimetric sensors in meat packaging signal the onset of spoilage through a change in color when volatile amines are present. These sensors are not only effective but also consumer-friendly and retailer-friendly. Systems have been able to attain detection limits as low as 0.5 ppm, thus demonstrating their potential to detect contamination at its earliest stages (Yu, Jing, & Cheng, 2023). Integration with Internet of Things (IoT) technology has amplified its capabilities with the wireless data transmission feature and central monitoring. An example of magnetic nanoparticle biosensors, which are integrated with poultry packaging, utilized LoRaWAN networks for the transmission of contamination alerts to the central servers. Such a system identified *Salmonella enterica* with 95% accuracy with much lesser dependency on human inspection and a much higher speed of decision-making (Musaev, 2024). Other nanosensors have been integrated into the packaging of fresh produce for monitoring humidity. Zinc oxide nanoparticles have been used in lettuce packaging to detect changes in moisture content; their use reduces the rate of spoilage by 30% because of early detection of conditions favorable for the growth of microorganisms (Smaoui et al., 2023).

Despite these opportunities, nanosensors also presented several challenges. Since their production costs are still very high and not very scalable, it is still hard to apply them to low-margin food products. Furthermore, they need durability and functionality under various conditions of storage and transportation. In this regard, a range of environmental factors can reduce the sensor performance due to temperature fluctuations, humidity, and long supply chain duration. With advancements in nanomaterial engineering and wireless communication, nanosensors will emerge even more practical and cheap. Self-powered nanosensors that can scavenge energy from ambient sources, such as light or motion, are being investigated to overcome power supply issues in remote and long-term monitoring applications. The integration of nanosensors with blockchain technology also provides a possibility for traceable contamination records, thereby enhancing food safety accountability throughout the supply chain. As these innovations continue to advance, nanosensors embedded in smart systems are promising for revolutionizing food safety monitoring, ensuring timely detection of contamination, and reducing food waste considerably. Particularly in developing regions, the deployment of low-cost, portable nanosensor systems—combined with mobile networks and blockchain—offers a scalable model for food safety monitoring with minimal infrastructure.

### 5.2 Big data and AI for predictive analytics in food safety

The convergence of nanotechnology, AI, and big data analytics holds huge promise to strengthen the monitoring of food safety. The coming together of all these technologies ensures rapid identification of pathogens and contaminants within food and food-related quality markers, promoting safe production, distribution, and consumption practices. This collaboration between the three is revolutionizing methodologies on food safety across agricultural and manufacturing sectors in the production line and within the health service sector. Nanotechnology has played a great role in ensuring food safety through highly sensitive nanosensors that can detect hazardous substances at extremely low levels. Such sensors are sensitive to the detection of pathogens within food products such as *Salmonella, E. coli,* and *Listeria* and also chemical pollutants like pesticides and toxins that ensure quality food and safety. For example, research was presented showing the use of sensors based on gold nanoparticles in detecting *E. coli* in contaminated water and food, with rapid and accurate detection even at a concentration of 10^4^ CFU/mL (Lin et al., 2024). AI, particularly machine learning (ML), further enhances the application of nanotechnology in food safety monitoring by enabling the analysis of large datasets from nanosensors and other food safety systems. AI algorithms can process data from various sources, including sensor networks, environmental data, and supply chain information, to identify patterns, predict risks, and optimize monitoring systems in real time. Research by Misra et al. (2020) found that AI-based systems integrated with nanosensors, improved the detection accuracy of pathogens in food samples and increased the speed of analysis by over 50%, as compared to conventional methods (Misra et al., 2022). Furthermore, AI-driven predictive models have been shown to reduce foodborne illness outbreaks by identifying potential contamination events before they escalate. Huang (2022) highlighted the application of deep learning models to predict the spread of pathogens in food supply chains, resulting in an 80% reduction in contamination incidents during pilot tests across multiple food production facilities (Huang et al., 2022).

Big data analytics is playing an important role in making the integration of AI and nanotechnology beneficial for food safety. Since the amount of data generated by nanosensors, AI systems, and supply chain operations is increasing, big data analytics processes and interprets these datasets in real time. Such systems would be useful for the researcher to examine massive data, thus enabling insight into a trend in food safety issues, emerging risks, and providing actionable recommendations on improvement in food production for those who produce, regulate, or consume food. Based on this study, it was clear that integration with big data analytics and AI and nanotechnology would better and more efficiently allow food safety management. For instance, it has been demonstrated that AI models developed on the basis of big data analytics can be used for real-time optimization of food quality control so that the pesticide residue detection accuracy exceeds 90% in agricultural produce (Das et al., 2024). The practical implementations of these integrated technologies underline their potential in changing the face of food safety monitoring systems. For example, Kuppusamy et al. (2021) explored integrating AI with nanosensors for early-stage pathogen detection in food production. It was established that foodborne illness outbreaks were reduced by 30%, indicating that AI-powered nanotechnology systems work efficiently in large-scale food safety operations (Kuppusamy et al., 2024). Furthermore, Lin et al. (2024) highlighted a nanotechnology-based system combined with big data analytics to monitor foodborne pathogens in the supply chain, reducing contamination risks by 40% through real-time data analysis (Lin et al., 2024). With synergistic interplay between nanotechnology, AI, and big data analytics, the pace of speed can be accelerated for food safety monitoring systems because it enhances speed, accuracy in contaminant detection, and high-risk prediction, as well as speed in response. All these aspects work towards scaling up and effective solutions toward real-time monitoring systems in all food supply chains. With advancements in sensor technologies, machine learning models, and data analytics, food safety in the future will depend on these integrations to ensure that people eating around the world remain safe and healthy. Thus, future emphasis must be placed on developing scalable, low-cost nanoparticle platforms that can be adopted even in resource-limited environments to ensure equitable access to food safety technologies

### 5.3 Blockchain for supply chain traceability in food safety

The integration of blockchain technology with nanotechnology-enhanced systems has transformed the traceability of food supply chains. This improved transparency, accountability, and safety. Utilizing the immutable and decentralized nature of blockchain, food manufacturers and regulatory bodies track all aspects of the supply chain from its origin to the point of consumption with unearthed accuracy (Ellahi et al., 2023). Nanosensors allow for the implementation of blockchain, thereby securing real-time information on potential contamination, storage conditions, and authenticity. Nanosensors can be incorporated into food packaging or processing environments to allow for the creation of instant data on parameters such as temperature, humidity, and microbial contamination. Such data uploaded in a system will create an immutable record that will be traceable over blockchain networks regarding the journey of food (Hang & Kim, 2019; Singh et al., 2021). For example, a pilot project on fish transport with blockchain-enabled nanosensors monitored the packed freshness of fish, helped reduce spoilage incidence by 25%, and minimized losses to food waste (Wang et al., 2022). Nanosensors for beverage production have been integrated with blockchain platforms to detect microbial contaminants at all stages of production, ensuring product integrity and reducing recall times by 50% (Kuswandi et al., 2017). The major threats to achieving global food safety are fraud caused by mislabelling or adulteration. Among the most reliable technologies for authenticating products and avoiding fraud are blockchain-based nanosensor systems. For example, molecular markers in olive oil can be identified using nanosensors, and this technology has been introduced into a blockchain network to track authenticity along all channels of distribution. It correctly detected mislabelled0 products 98% of the time, which increased customer confidence (Frigerio et al., 2024). In the dairy value chain, blockchain sensors track milk quality from the farm to the retail stage. They can detect the occurrence of contamination and tag it with specific suppliers. The traceability decreased the contamination investigation time by 60%. This means that cost savings and the prevention of potential outbreaks will be realized (Khanna et al., 2022). It uses blockchain technology and maintains an openly accessible log of all the occurrences within a supply chain.

Regulators determine how safe the handling of such products is, as per the information in the blockchain. This reduced the number of physical audits needed along with the certification process. Nanosensors have been utilized in blockchain-based systems for poultry to monitor the temperature during transit. The specified conditions were differentiated automatically, which expedited correction and compliance with the rules (Biswas et al., 2024; Poeta et al., 2023). Nevertheless, the integration of blockchain technology with nanotechnology-based food safety systems remains an issue. For example, the implementation cost is a concern for small-scale producers. Some issues that need to be solved include the interoperability between different blockchain platforms and data security. Standardized frameworks for implementation are determined through collaborations between education and stakeholders. The reality of blockchain in the food safety realm is the application of blockchain in AI-driven analytics and IoT-enabled monitoring systems. These combined technologies will not only enhance traceability but also enable predictive insights and ensure proactive interventions against contamination risks. With more cost-effective blockchain solutions, their use in the food industry will advocate for more transparency, safety, and consumer confidence.

### 5.4 Microbial sensors for real-time monitoring and contamination control

Microbial sensors have emerged as powerful tools for ensuring food safety through early detection and real-time monitoring of microbial contamination. These systems can be classified based on their applications: (i) source-level detection (e.g., during raw material intake), (ii) processing-level monitoring (e.g., on surfaces or equipment), and (iii) storage-level monitoring to detect microbial shifts during distribution. Several advanced platforms have been developed, including electronic nose (e-nose) systems, fluorescent-based DNAzyme sensors, and optical biosensors, which offer fast, non-destructive, and label-free monitoring. For example, Sentinel Wraps—transparent, flexible packaging embedded with DNAzyme probes—have shown real-time fluorescence detection of *E. coli* in meat and juice products, with limits of detection as low as 10^3^ CFU/mL (Yousefi et al., 2018). Similarly, MOX-NW-based electronic noses have been used to detect microbial spoilage in soups and tomato paste within 24 hours (Sberveglieri et al., 2014). During food processing, low-cost microbial test kits have shown promise for surface hygiene monitoring, offering a rapid and affordable approach for routine safety checks (Saad et al., 2018). For storage and transport, smart sensors that detect microbial metabolites (e.g., amines) in packaged foods have gained attention for tracking spoilage progression (Khan et al., 2023). In addition to biosensors and real-time monitoring systems, advanced molecular technologies such as Direct Analysis in Real Time Mass Spectrometry (DART-MS) and real-time fluorescence quantitative PCR (qPCR) offer valuable tools for food safety detection.

DART-MS is an ambient ionization technique that enables rapid, in-situ analysis of food samples with little or no sample preparation. It operates under atmospheric pressure and is especially effective when combined with mass spectrometry for high-throughput screening of contaminants, residues, and adulterants. DART-MS has been successfully applied to detect chemical residues in meat, grains, packaging materials, and dairy, offering an environmentally friendly and fast alternative to conventional LC-MS/GC-MS methods (Wang, 2024), (Zhang et al., 2020). Moreover, DART has shown over 90% detection accuracy for trace chemical contaminants in complex matrices with total analysis times under 1 minute (Khaled et al., 2020). On the other hand, real-time fluorescence qPCR remains a gold standard for DNA-based detection of foodborne pathogens and adulterants. It offers excellent sensitivity and specificity, particularly useful in meat species identification and microbial quantification. Unlike DART, which focuses on chemical profiling, qPCR targets nucleic acid sequences and can detect as few as 10 gene copies per reaction. However, qPCR generally requires skilled personnel, precise thermal cycling equipment, and has limited portability for field-based applications. Both DART and qPCR represent powerful yet complementary tools—DART excels in non-destructive chemical screening with minimal prep, while qPCR is unmatched in genetic identification and quantification. Future diagnostic systems may benefit from combining these techniques with biosensors for broader, faster, and more integrated food safety monitoring. Looking forward, microbial sensors are expected to become more integrated with IoT and blockchain, enabling real-time cloud-based contamination alerts, predictive analytics, and traceability across the supply chain.

## 6 Regulatory landscape and commercial viability

### 6.1 Regulatory standards: addressing challenges and establishing global frameworks

For Nanoparticles in Food Safety

The adoption of nanoparticle-based systems in the food industry is intrinsically tied to the development of robust and harmonized regulatory standards. At present, the global guidelines established by the FDA, EFSA, and WHO remain fragmented, thereby creating significant hurdles for the commercialization and application of such advanced technologies (as depicted in Table 3). In the United States, no specific regulation by the FDA defines the Generally Recognized as Safe (GRAS) list of food contact materials regarding the specific physicochemical properties of nanoparticles, thus creating such critical gaps in oversight in the country (Commissioner, 2024a, Commissioner, 2024b; Onyeaka, Passaretti, Miri and Al-Sharify, 2022a, Onyeaka, Passaretti, Miri and Al-Sharify, 2022b). Similarly, the EFSA requires detailed evaluations of engineered nanomaterials in food but lacks standard methodologies for assessing nanoparticle toxicity to create inconsistent regulation decisions (EFSA, 2021). Key regulatory concerns include the potential toxicity risks posed by nanoparticles due to their small size, high surface area, and unique reactivity. Research has demonstrated that materials such as silver nanoparticles and titanium dioxide may induce oxidative stress and inflammatory responses in human cells, raising concerns regarding their safety in food applications (Martirosyan & Schneider, 2014). Moreover, environmental issues are attributed to nanoparticles that find their way into ecosystems, where they accumulate and threaten aquatic and terrestrial life. There is currently no comprehensive regulatory framework used to assess these effects, making it challenging to approve nanoparticle-enabled systems (Park & Yeo, 2016). The lack of harmonized international standards amplifies these challenges and discourages innovation, thus creating a fragmented regulatory environment. WHO has underlined the need for urgent, collaborative risk assessments, emphasizing gaps in data and methodologies for evaluating nanoparticle behaviour in food matrices and during long-term exposure. These barriers must be overcome through coordinated efforts such as the establishment of uniform testing protocols and shared data repositories that facilitate comprehensive assessments of nanoparticle safety. Advances in nanotoxicology and computational modeling have promised improved prediction of nanoparticle interactions and streamlined regulatory reviews. Clear and consistently strong global standards are critical not only for ensuring the safety of applications of nanoparticles but also for improving innovation that would allow for market growth. Despite these challenges, there have been several successful cases of nanoparticle-based technologies gaining regulatory approval. For example, gold nanoparticle-based biosensors for aflatoxin B1 have achieved EU-compliant sensitivity in real food matrices (Liu et al., 2023). While direct regulatory approval for systems such as NanoCeramic or InnovaPrep is not publicly confirmed in scientific literature, these technologies represent the growing class of translational nanodiagnostics advancing toward commercialization. Strong early validation, risk-based safety reviews, and collaborative engagement with agencies like MFDS and USDA are key to accelerating their regulatory pathway. Collaboration between regulatory bodies, academic researchers, and industry stakeholders is very important in extracting every possible revolutionary benefit from nanoparticle-based systems in food safety while maintaining public trust and environmental sustainability. To overcome these regulatory barriers, several solutions have been proposed. First, the harmonization of international guidelines through joint task forces (e.g., WHO–FAO Codex committees) can facilitate consistent nanoparticle evaluation across borders (Souto et al., 2024). Second, standardized toxicological testing frameworks, such as in vitro high-throughput assays and predictive computational models (e.g., Quantitative Structure–Activity Relationship models), can improve risk prediction and regulatory throughput. Third, the creation of public–private innovation hubs can allow industry, academia, and regulators to co-develop nanoparticle characterization protocols, fostering transparency and speeding up approval timelines. Fourth, life-cycle risk assessments—from production to disposal—can help address both safety and environmental sustainability. Lastly, increased funding for nanotoxicology databases and global data-sharing platforms can close knowledge gaps and build public confidence. These strategies, if adopted collaboratively, can pave the way for safer and faster adoption of nanoparticle-based innovations in food safety (Schoonjans et al., 2023).

**Table 3:** Overview of regulatory focus, key concerns, current efforts, and references by leading food safety authorities on nanotechnology in food.

Organization	Regulatory Focus	Key Concerns	Current Efforts	References
**FDA (United States Food and Drug Administration)**	Safety evaluation of food contact materials; GRAS designation for nanomaterials.	Lack of explicit regulations for nanoparticles; limited data on human toxicity.	Guidelines for nanoparticle characterization; encouraging industry submissions for novel materials.	(Commissioner, 2024)
**EFSA (European Food Safety Authority)**	Risk assessment of engineered nanomaterials; physicochemical characterization and toxicology studies.	Absence of harmonized risk assessment protocols; inconsistent evaluations across EU member states.	Developing a harmonized risk assessment framework for nanotechnology in food applications.	(“EFSA | Science, safe food, sustainability,” 2021)
**WHO (World Health Organization)**	Global health guidance on nanotechnology in food; emphasis on long-term exposure risks.	Gaps in data for environmental and human health risks; limited methodologies for nanoparticle behavior.	Collaborations with academic and industrial bodies to address knowledge gaps in nanotoxicology.	(“World Health Organization (WHO),” n.d.)
**ISO (International Organization for Standardization)**	Standardization of testing methodologies for nanomaterials in food systems.	Inadequate coverage of nanospecific guidelines in food applications; challenges in universal standards.	Initiatives for international standardization of nanotechnology safety protocols.	(“ISO - International Organization for Standardization,” 2024)
**FAO (Food and Agriculture Organization)**	Global collaboration on food safety; frameworks for sustainable nanotechnology applications.	Limited emphasis on nanotechnology's role in sustainable agriculture and food systems.	Promoting international collaboration on regulatory best practices and sustainable nanotechnology.	(FAO, n.d.)
**FSANZ (Food Standards Australia New Zealand)**	Review of nanomaterials in food additives and food contact materials.	Limited evidence on nanoparticle bioavailability and toxicology in food applications.	Regular evaluations of novel nanomaterials to ensure consumer safety and environmental protection.	(“Homepage | Food Standards Australia New Zealand,” n.d.)
**FSSAI (Food Safety and Standards Authority of India)**	Food safety and quality monitoring for nanomaterials in food and agriculture.	Lack of domestic testing infrastructure; limited regulatory expertise in nanotechnology.	Building partnerships for research on nanotechnology applications in food safety and nutrition.	(“FSSAI,” n.d.)
**Health Canada**	Risk assessment of engineered nanomaterials in food and packaging.	Unclear risk thresholds for nanoparticle exposure; limited long-term data.	Published guidelines for nanomaterial characterization and testing in food safety applications.	(Canada, 2020)
**JECFA (Joint FAO/WHO Expert Committee on Food Additives)**	Expert advice on food additives and contaminants, including nanomaterials.	Lack of specific data on nanomaterial toxicity; limited understanding of their behavior in food systems.	Coordinating research projects and standardizing methodologies for evaluating food nanomaterials.	(“JECFA | Food safety and quality | Food and Agriculture Organization of the United Nations,” n.d.)
**OECD (Organisation for Economic Co-operation and Development)**	Safety assessments of nanomaterials in global food markets.	Variability in regulatory approaches across member countries; inconsistent definitions of nanomaterials.	Developing internationally accepted test guidelines and frameworks for nanotechnology in food.	(“Better policies for better lives,” n.d.)

### 6.2 Current market trends: opportunities and barriers in nanoparticle-based detection systems

Nanoparticle-based detection systems are being introduced as the next big deal in food safety, bringing about unprecedented sensitivity, speed, and versatility over conventional methods. These systems utilize the distinctive physicochemical properties of nanoparticles, including high surface area, optical tunability, and functionalization capacity, for highly precise detection of foodborne pathogens. Gold and magnetic nanoparticles are among the leading players in this arena, allowing rapid and highly specific pathogen detection. For example, based on gold nanoparticles, multiplex PCR assays allow one to simultaneously determine pathogens at picogram levels of DNA concentration achieving results in minutes without undergoing lengthy sample preparation steps such as those provided by traditional methods (Du et al., 2020). It definitely brings huge benefits of decentralized testing, especially within a resource-limited setting, or even in a field setting, where people crave portable and rapid diagnostic tools. Probably one of the most exciting aspects of the nanoparticle-based system is their capability for multiplex detection, which has ever been more in demand, given the growing requirement of food safety regulations for performing a much more profound screening procedure. Some technologies, such as mesoporous silica nanoparticle-enhanced microarrays and aptamer-based biosensors, allow for the detection of several pathogens in one test, saving time and money compared to the sequential approach. These technologies have some special relevance to complicated matrices such as processed meat, dairy, or seafood because the concentrations of these pathogens are often very low and the results obtained through classical methods tend not to be consistent (Hormsombut et al., 2024). Even better integration has been accomplished by combining nanoparticles with such advanced platforms as surface-enhanced Raman spectroscopy or quantum dots. Such enhanced capabilities in the diagnosis of food safety in respect to real-time monitoring with detection sensitivity that was unique through reaching up to 10 CFU/mL to those pathogens, such as *E. coli,* on such a real-world sample of food (Schmoldt et al., 1975).

Despite all these advantages, there are several significant challenges in the way toward using these nanoparticle detection systems more widely. Among these is the scaling and yet low-cost production level; gold nanoparticles are very cost expensive materials, and yet one may not be afforded a sufficient amount of their access is restricted especially towards less- and middle-class developing nations. Moreover, the performances of these systems in complex food matrices are inconsistent because of interfering components such as fats, proteins, and carbohydrates, which may interfere with nanoparticle-pathogen interaction, necessitating further optimization and validation for reliable results across various sample types (Rodriguez-Quijada et al., 2020). The second critical barrier is the absence of standardized regulatory frameworks for nanoparticle-based systems. The traditional methods such as PCR and culture-based assays have well-defined regulatory pathways. However, nanotechnology introduction required extended safety assessments, performance benchmarks, and cross-border harmonization. This absence of such frameworks was a significant hindrance to commercialization and slow adoption in the global market (Shen et al., 2021). Consumer perceptions also contribute to the adoption barriers. There are still public concerns regarding nanoparticle safety and toxicity, although there is enough evidence that these materials are safe for use in diagnostic applications. For the food industry, integration of nanoparticle-based systems required high investment, not only in the purchase of equipment but also in training personnel and making the processes compliant with new regulatory requirements (Igberaese Clinton Festus-Ikhuoria et al., 2023; Onyeaka et al., 2022). The demand for innovative food safety solutions, driven by regulatory pressures and the global food safety testing market projected to reach $24 billion by 2026, continues to grow. Growth in this area points out the need to overcome such challenges through collaboration between researchers, regulatory agencies, and industry stakeholders to streamline commercialization pathways and improve affordability. Nanoparticle-based detection systems present incredible opportunities to revolutionize diagnostics for food safety due to superior sensitivity, multiplex capability, and portability (“Food Safety Testing Market Share, Forecast | Growth Analysis & Opportunities,” n.d.). However, their success hinges on addressing critical barriers such as scalability, cost, and regulatory standardization. With continued advancements in technology and supportive policy frameworks, these systems are well-positioned to become a cornerstone of global food safety strategies, ensuring healthier and safer food for all. Lastly Table 4, lists commercially available nanoparticle-based detection systems for foodborne pathogen detection.

**Table 4:** Overview of commercially available nanoparticle-based detection systems for foodborne pathogen detection.

Technology/Product Name	Company/Provider	Nanoparticle Type	Target Pathogen	Detection Method	Key Features
**Veriflow® Assay**	Invisible Sentinel (now part of BioMérieux)	Gold nanoparticles	*Salmonella*, *E. coli O157:H7*, *Listeria monocytogenes*	Lateral flow immunoassay with PCR	Combines PCR amplification with nanoparticle visualization for sensitive, rapid detection.
**3M™ Molecular Detection System**	3M	Magnetic nanoparticles	*Salmonella*, *Listeria*, *E. coli*	Isothermal DNA amplification	Uses magnetic nanoparticles to capture target DNA, enabling high-throughput pathogen screening.
**NanoMAG Pathogen Detection Kits**	Nanomagnetic Instruments	Magnetic nanoparticles	*E. coli*, *Salmonella*, *Listeria*	Immuno-magnetic separation	High-speed separation of pathogens from complex food samples for accurate analysis.
**BioFilm Detection Sensor (BFS-10)**	ZP Biomedical	Gold nanoparticles	Biofilms of foodborne pathogens	Colorimetric biosensing	Detects biofilms of multiple pathogens on surfaces using gold nanoparticle-based biosensors.
**PathogenDx DetectX-R**	PathogenDx	Quantum dot nanoparticles	*E. coli*, *Salmonella*, *Listeria*	Microarray with fluorescence detection	Uses nanotechnology in DNA microarrays for rapid and highly sensitive multiplex detection.
**ELISAONE® Pathogen Tests**	Avacta Life Sciences	Gold nanoparticles	*Listeria monocytogenes*, *E. coli*	ELISA with nanoparticle enhancement	Gold nanoparticles enhance signal strength for rapid and quantitative detection.
**Qplex™ Food Safety Test**	Quansys Biosciences	Gold nanoparticles	*Salmonella*, *Listeria*, *E. coli O157:H7*	Multiplex ELISA	Allows simultaneous detection of multiple pathogens in a single sample.
**NanoLogix BNP Kits**	NanoLogix	Nanoparticles (unspecified)	*Salmonella*, *E. coli*, *Listeria*	Rapid detection and culturing	Utilizes nanoparticle-based methods for real-time pathogen detection with reduced incubation times.
**Envision™ Immunoassay System**	PerkinElmer	Gold nanoparticles	Various pathogens	Immunoassay	Nanoparticles enhance antibody-antigen signals for sensitive pathogen identification.
**OptiSense® Pathogen Detection**	NanoBioMatters	Gold nanoparticles	*Salmonella*, *Listeria*, *E. coli*	Plasmonic biosensing	Detects pathogens via localized surface plasmon resonance (LSPR) changes in nanoparticles.
**BIOTECON Foodproof® Detection Kits**	BIOTECON Diagnostics	Magnetic nanoparticles	*Salmonella*, *Listeria monocytogenes*	PCR with nanoparticle enhancement	Rapid sample preparation and detection in less than 24 hours.
**Vidia Nanobiosensor Kits**	Vidia Technologies	Gold nanoparticles	*E. coli*, *Salmonella*, *Listeria*	Surface plasmon resonance (SPR)	Portable, real-time pathogen detection in food processing facilities.
**FoodScan® Pathogen Analyzer**	NanoSense	Magnetic nanoparticles	*E. coli*, *Salmonella*	Magneto-resistive biosensing	Uses magnetic nanoparticle biosensors for rapid detection in liquid samples.
**Magbio Pathogen Detection Kits**	Magbio Genomics	Magnetic nanoparticles	*Salmonella*, *E. coli*, *Campylobacter*	Magnetic bead-based nucleic acid extraction	Focuses on nucleic acid extraction for accurate downstream molecular detection.

## 7 Conclusion

Nanoparticle-based detection systems are a much-needed development in the area of food safety, answering an age-old deficiency using conventional pathogen detection methodology. This much-needed development for the food safety area was in answering the age-old deficiency by applying the conventional methodology in the pathogen detection approach. These nanoparticle-based detection systems have manifested higher sensitivity and specificity, thus making them versatile enough to detect different types of pathogens that come from diverse food categories, including dairy products, seafood, fresh produce, and processed foods. This has ensured strong performance under adverse conditions and provides the basis for further development and improvement of food safety protocols ranging from functionalization and hybrid platforms to real-time monitoring. In intelligent nanotechnology systems, including nanosensors in packaging and traceability through blockchain, significant paradigm shifts can be anticipated in food safety management. This ensures better real-time detection of contamination and enhances transparency at all supply levels to reduce waste and increase consumer confidence. However, massive integration poses severe challenges, such as high production costs, vague regulatory systems, and public concern over the safety of applying nanotechnology to food items. Regulatory agencies should establish strict and standardized rules that help stakeholders visualize the toxicity and environmental concerns of nanoparticles. Future initiatives will focus on joint efforts by researchers, policymakers, and industry heads to upgrade nanoparticle detection techniques. Therefore, the course of detection technologies must be dictated by the commercialization of nanoparticles with lower production costs, effective regulatory measures, and education programs. Continued innovation and strategic implementation of nanoparticle-based detection systems can also help revolutionize food safety and provide safer and more sustainable food systems for everyone in this world. Looking ahead, the real impact of nanoparticle-based detection systems will depend on their successful translation into scalable, cost-effective, and regulatory-compliant solutions. The development of portable, real-time biosensors and integration into smart packaging and blockchain systems shows high promise for enhancing traceability and consumer trust. However, large-scale adoption will require advances in mass production, material safety validation, and interdisciplinary collaboration between regulators, researchers, and industry. With continued innovation and harmonized global standards, nanoparticle-enabled diagnostics can move from lab to market, revolutionizing the future of food safety.

## CRediT authorship contribution statement

**Himanshu Jangid:** Writing – original draft. **Mitali Panchpuri:** Writing – original draft. **Joydeep Dutta:** Writing – original draft. **Harish Chandra Joshi:** Writing – original draft, Resources. **Maman Paul:** Writing – review & editing. **Arun Karnwal:** Writing – original draft, Resources. **Akil Ahmad:** Visualization, Writing – original draft. **Mohammed B. Alshammari:** Writing – review & editing, Validation, Conceptualization. **Kaizar Hossain:** Conceptualization, Writing – review & editing, Supervision. **Gaurav Pant:** Conceptualization, Supervision, Writing – review & editing, Validation. **Gaurav Kumar:** Conceptualization, Validation, Writing – review & editing.

## Declaration of competing interest

The authors declare that they have no known competing financial interests or personal relationships that could have appeared to influence the work reported in this paper.

## Data Availability

No data was used for the research described in the article.

## Appendix A Supplementary data

Supplementary material Image 1
