# Supplementary material for: Nanoparticle-based detection of foodborne pathogens: Addressing matrix challenges, advances, and future perspectives in food safety
Source: Food Chem X. 2025 Jun 25;29:102696. doi: 10.1016/j.fochx.2025.102696 (PMC12266482; doi:10.1016/j.fochx.2025.102696)
Supplement: Supplementary file 1 — Supplementary material [file mmc1.pdf]

Authorship change request

Important information. Please read before completing this form.
This form is to request any change in authorship (additions, removals, or reordering) of a submitted manuscript, including changes in corresponding authors. This form should not be used for changes requested *after* publication or for name changes or corrections.

Prior to completing this form, all authors should carefully review the 'duties of authors' section of the Elsevier publishing ethics policy, and in particular, the sections on:

- Authorship of the paper
- The use of generative AI and AI-assisted technologies in scientific writing

Please also carefully review the journal's guide for authors (this might also be referred to as 'instructions for authors') as some journals may have additional authorship criteria (e.g., the ICMJE guidelines for authorship).

The publisher and editor cannot investigate or mediate any authorship disputes. If you are unable to obtain agreement from all authors, including those you intend to remove, we recommend seeking guidance from your institution. We will not consider your change request and will not proceed with the publication of your manuscript until all outstanding authorship disputes are resolved.

In order for the request to be considered, this completed form should be submitted in Editorial Manager with the cover letter for your revision.

Section 1. Submission information

To be completed by the corresponding author.

**Submission information**	
Journal title	Food Chemistry: X
Manuscript number	FOCHX-D-25-00227
Manuscript title	Nanoparticle-Based Detection of Foodborne Pathogens: Addressing Matrix Challenges, Advances ,and Future Perspectives in Food Safety

**Change(s) requested** (indicate as appropriate)	
☑ Add new author(s)	☐ Remove author(s)
☐ Change the corresponding author	☐ Change the order of authors

Section 2. Author(s) added or removed

Complete one table for each author to be added or removed. Please include as much detail as possible in the "Reason for change" section so that we can evaluate if the change is necessary. At a minimum, this should include an explanation for why the change is being requested and why the author was/was not included in the original author list.

If the form is incomplete, or the reasons provided are insufficiently detailed or do not address the points above, your request will be denied.

2.1 Author information

Given/first name(s)	Maman
Family/last name	Paul
Email address	mamanpaul@gmail.com
Institution	Department of Physiotherapy, Guru Nanak Dev University, Amritsar, Punjab, India
Change(s) requested (indicate as appropriate)	☑ Add new author ☐ Remove author ☐ Make the corresponding author

Individual contributions per CRediT Contributor Roles Taxonomy (complete for author additions only)

☐ Conceptualization	☐ Data curation	☐ Formal analysis
☐ Funding acquisition	☐ Investigation	☐ Methodology
☐ Project administration	☐ Resources	☐ Software
☐ Supervision	☐ Validation	☐ Visualization
☐ Writing – original draft	☑ Writing – review & editing	

Reason for the change

Due to sudden health issue of Dr. Harish Chandra Joshi and unavability of 3 authors, I (Dr. Gaurav Pant - Corresponding Author) have to take help of Dr. Maman Paul. The author actively participated in writing and restructuring the manuscript after revision.

Section 3. Author order and agreement

Provide the author list in the order that you would like it to be published.

The form must be signed individually by each author, including any added/removed authors. In cases of multi-author collaborative or consortia groups the corresponding author must sign on behalf of the group.

While manual signatures are acceptable, we highly encourage the use of electronic signature software (DocuSign, Adobe Sign, HelloSign, or similar) with valid e-signatures. These signatures should reflect your institutional information and email, as provided in the author list below. Typed signatures or images of signatures will not be accepted.

By signing this form all authors agree:

1) that they have read and acknowledge the publishing ethics policies linked in the "Important Information" section of this form;
2) agree to the addition and/or removal of the authors listed in section 2 and to the revised order of the author list in this section 3, and;
3) that all information provided accurately reflects the authorship of the article.

Agreement of removed author(s)

Full name	Email address	Signature	Date

Proposed author list

Order	Full name	Email address	Signature	Date
01	Himanshu Jangid	himanshujangid443@gmail.co	Himanshu	22/3/25
02	Mitali Panchpuri	mitali.panchpuri44@gmail.con	[illegible]	22/3/25
03	Joydeep Dutta	jdutta08@gmail.com	Joydeep	22/03/25
04	Harish Chandra Joshi	joshiharish86@gmail.com	[illegible]	22/3/25
05	Maman Paul	mamanpaul@gmail.com	M Paul	22/3/25
06	Arun Karnwal	arunkarnwal@gmail.com	[illegible]	21/3/25
07	Akil Ahmad	akil aj.ahmad@psau.edu.sa	[illegible]	21/3/25
08	Mohammed B Alshammari	m.alshammari@psau.edu.sa	[illegible]	21/3/25
09	Kaizar Hossain	kaizar.kaizar@gmail.com	K. Hassain	22/3/2025
10	Gaurav Pant	rgauravpant@gmail.com	[illegible]	21/3/2025.

Proposed author list

Order	Full name	Email address	Signature	Date
11	Gaurav Kumar	gauravkr01@gmail.com	[illegible]	22-03-25
12				
13				
14				
15				
16				
17				
18				
19				
20				
21				
22				
23				
24				
25				
26				
27				
28				
29				
30				
31				
32				
33				
34				
35				

*Add additional page(s) as needed.
